# Intraspecific antagonism through viral toxin encoded by chronic *Sulfolobus* spindle-shaped virus

**DOI:** 10.1098/rstb.2020.0476

**Published:** 2022-01-17

**Authors:** Samantha J. DeWerff, Changyi Zhang, John Schneider, Rachel J. Whitaker

**Affiliations:** ^1^ Department of Microbiology, University of Illinois at Urbana-Champaign, Urbana, IL, USA; ^2^ Carl R. Woese Institute for Genomic Biology, University of Illinois at Urbana-Champaign, Urbana, IL, USA

**Keywords:** archaea, chronic viruses, archaeal toxins, virus–host mutualism

## Abstract

Virus–host interactions evolve along a symbiosis continuum from antagonism to mutualism. Long-term associations between virus and host, such as those in chronic infection, will select for traits that drive the interaction towards mutualism, especially when susceptible hosts are rare in the population. Virus–host mutualism has been demonstrated in thermophilic archaeal populations where *Sulfolobus* spindle-shaped viruses (SSVs) provide a competitive advantage to their host *Sulfolobus islandicus* by producing a toxin that kills uninfected strains. Here, we determine the genetic basis of this killing phenotype by identifying highly transcribed genes in cells that are chronically infected with a diversity of SSVs. We demonstrate that these genes alone confer growth inhibition by being expressed in uninfected cells via a *Sulfolobus* expression plasmid. Challenge of chronically infected strains with vector-expressed toxins revealed a nested network of cross-toxicity among divergent SSVs, with both broad and specific toxin efficacies. This suggests that competition between viruses and/or their hosts could maintain toxin diversity. We propose that competitive interactions among chronic viruses to promote their host fitness form the basis of virus–host mutualism.

This article is part of the theme issue ‘The secret lives of microbial mobile genetic elements’.

## 1. Introduction

Viruses of microbes play key roles in shaping the population dynamics of their host. This can be due to antagonistic interactions, such as those with lytic viruses, or more mutualistic interactions, in which the host is benefited [1,2]. Such benefits can include growth advantages, for example by viruses that encode auxiliary metabolic genes [3–5], or competitive advantages in which the infected hosts are able to outcompete their uninfected counterparts such as through the production of intraspecific toxins [6].

Microbes produce a broad array of proteinaceous toxins that antagonize closely related competitor strains, many of which are carried on mobile genetic elements, including prophages [7,8]. While some toxins are important virulence factors that interact with other organisms, many microbes also carry polymorphic toxin systems that target closely related strains [9,10]. These toxin systems play a critical role in ecological interactions and in establishing or mediating interactions in complex environments; for example, human microbiome-associated bacteria of the Firmicutes carry MuF polymorphic toxins, which are encoded on prophages [11].

Toxin systems of archaea are not as well studied as their bacterial counterparts but are predicted to be just as diverse and numerous [8]. In the archaeal order Sulfolobales, only two toxin-associated genes, the products of which form ‘sulfolobicin’, have been characterized to date [12]. No toxins have been described on viruses of archaea. Infection with viruses has been shown to induce host toxin–antitoxin systems [13–16]. In their carrier (non-induced) state, very few viral genes are expressed, the majority of which are predicted to encode structural proteins [16].

We previously demonstrated that in *Sulfolobus islandicus*, chronic infection with *Sulfolobus* spindle-shaped virus 9 (SSV9) provides a competitive advantage over uninfected strains, including those immune to SSV9, while imposing only a minor growth cost [17]. SSVs are chronic viruses that infect *Sulfolobus* species and reproduce by budding from host cells, without lysis [18]; they, therefore, transmit both vertically and horizontally. All SSVs encode an integrase, allowing them to insert into the host chromosome as a provirus, though this is not an essential function [19–21]. Virions are fusiform and contain an approximately 17 kb circular dsDNA genome [20,22]. The competitive advantage associated with SSV9 infection is due to the ability of chronically infected hosts to kill uninfected hosts, even when initially rare in the population [17]. Furthermore, this is not unique to SSV9–SSV13, and SSV17, also isolated from Kamchatka, Russia, as well as SSV11, isolated from Yellowstone National Park, USA, were also found to confer this phenotype. The Kamchatka-derived SSV14 did not show a killing phenotype [17]. It was also shown previously that SSV-conferred toxicity is mediated by a proteinaceous killing factor produced by the chronically infected cell [17]. The infected cell is resistant to the effect of the factor it produces, but we hypothesized that the production of this factor is responsible for the cost of infection.

In this study, we identify genes in several SSVs that confer the previously described competitive mutualistic phenotype. We use RNA sequencing to determine highly expressed viral genes in the chronically infected state and clone these genes into a *Sulfolobus* overexpression vector to test for toxin activity. We show that though the specific toxin protein varies among SSVs, it is encoded in the same position in the viral genome. Finally, challenge of chronically infected strains with different toxins suggests there may be competition among hosts mediated by their mutualistic viral infections.

## 2. Results

### (a) Viral transcription in chronically infected state SSV9 and re-annotation of ORF B252

**Figure 1.**
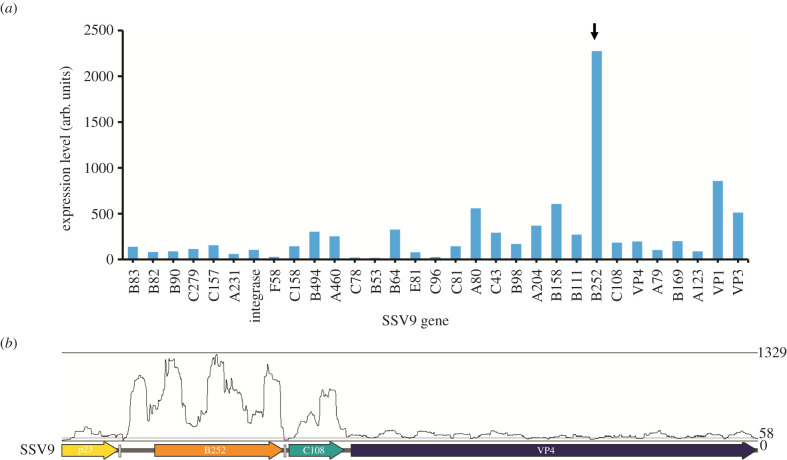
Expression of viral genes in chronically SSV9-infected strain. *Sulfolobus islandicus* RJW002Δ*cas6* was infected with SSV9, and then subjected to RNA-seq analysis. (*a*) Expression values are calculated by Rockhopper and are representative of three biological replicates. Black arrow points to putative toxin gene of interest. (*b*) Transcription of SSV9 B252 and C108 indicated by coverage of mapped RNAseq reads.

To identify toxin candidate genes in SSV9, we assessed viral transcription in chronically infected strains grown to mid-log phase with ongoing viral and toxin production. As shown in figure 1*a*, the hypothetical gene annotated as ORF B252 is highly transcribed in the infected state along with the genes encoding viral coat proteins VP1 and VP3. B252 is encoded on the T3 transcript along with one other hypothetical gene oriented in the same direction, ORF C108 [23,24]. These are followed by the core SSV gene, VP4, predicted to encode viral tail fibres (figure 1*b*) [25].

Based on the transcription start site and homologues encoded by other SSVs, we identified a mis-annotation of B252 in the RefSeq genome and extended it from 252 to 310 amino acids (figure 1*b*, electronic supplementary material, figures S1 and S2) to include a membrane trafficking signal sequence as predicted by SignalP5.0 [22]. We thus re-annotated this gene as SSV9 B310 for future analysis. The corrected gene also codes for an N-terminal signal peptide (see electronic supplementary material, figure S1).

Previously characterized archaeal toxins frequently exist as a two to three gene neighbourhood [8,12]; for example, the single characterized sulfolobicin consists of two proteins with signal peptides [12]. In addition, other predicted toxins in the Sulfolobales contain one or more transmembrane domains [8]. In SSV9, the gene C108 is located directly downstream of B310 on the same transcript and contains three putative transmembrane domains predicted by Phobius [26] (electronic supplementary material, figure S2); it is also expressed in infected cells (figure 1), although not as highly as B310. We, therefore, hypothesized that SSV9 B310 and C108 encode a viral toxin and antitoxin, respectively, that together mediate the killing phenotype.

### (b) B310 encodes a toxin that inhibits uninfected cells and protects infected ones

**Figure 2.**
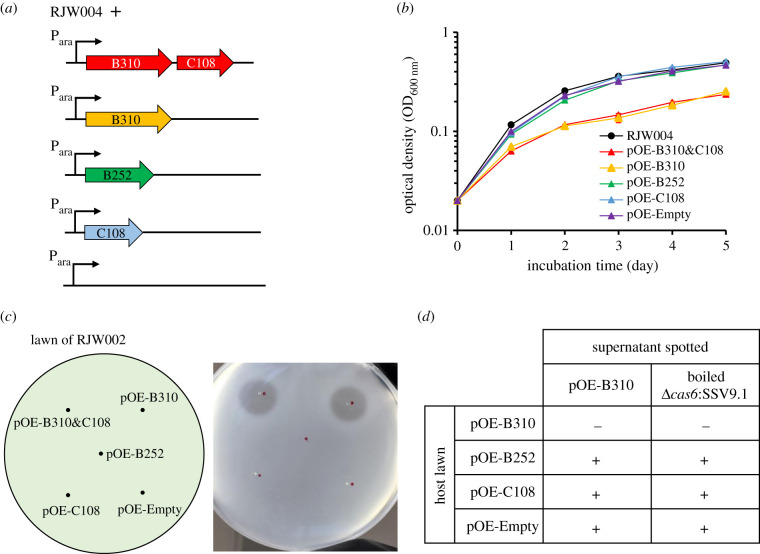
Strains expressing B310 show the toxin phenotype. (*a*) Uninfected RJW004 strain was transformed with plasmids that contained either both B310 and C108, B310 alone, the originally annotated B252 alone, C108 alone or empty plasmid under the control of inducible arabinose promotor. (*b*) Growth curve showing that there is a cost to carrying plasmids with the B310 gene. Strains were grown in induced conditions in the presence of arabinose as the carbon source in the medium. Error bars show mean results ± standard error of the means (s.e.m.) (*n* = 3). (*c*) Supernatants from RJW004 strains with a plasmid encoding B310 can produce a zone of inhibition on uninfected strain RJW002. (*d*) Supernatants from B310 overexpression strain or boiled supernatant from the chronically SSV9-infected strain were spotted onto lawns of the RJW004 strain carrying pOE-B310, pOE-B252, pOE-C108 and pOE-Empty. A plus sign indicates the presence of a zone of inhibition and a negative sign indicates the absence of a zone of inhibition.

To test for toxicity, each of the candidate genes (B310, C108 and the two genes combined), as well as the originally annotated gene B252 were cloned into a *Sulfolobus* expression vector pSeSD-SsoargD (hereafter pOE-Empty) under the control of an arabinose-inducible promoter (figure 2*a*). The resulting overexpression vectors pOE-B310, pOE-C108, pOE-B310&C108 and pOE-B252 were transformed into *S. islandicus* RJW004, and the strains thus generated were grown in inducing conditions with arabinose as carbon source (figure 2*b*). Cell-free culture supernatants collected from overexpression strains after 48 h of growth in inducing conditions were spotted onto *S. islandicus* RJW002 to test for the presence of a zone of inhibition, indicative of the toxic phenotype [17]. Growth curves of the engineered strains were also obtained to detect any changes in growth due to the expressed genes. As shown in figure 2*b*,*c*, strains containing pOE-B252, pOE-C108 or pOE-Empty showed no growth reduction in culture and their supernatants did not produce zones of inhibition. By contrast, strains carrying pOE-B310 and pOE-B310&C108 had a significant reduction in growth rate, with doubling times of 19.2 and 18.9 h in exponential phase, respectively, compared with 13.0 h for wild-type (*p* < 0.005). Supernatants taken from these cultures produced zones of inhibition when spotted onto lawns of RJW002 (figure 2*c*). These data show that the presence of the B310 protein product causes a toxic effect in *S. islandicus*, completely inhibiting the growth of RJW002 and slowing the growth of the producing strain; this implies that it is the gene responsible for the toxic phenotype conferred by SSV9.

Though growth retardation was seen in the B310 overexpression strains, cells expressing the putative toxin were not killed, suggesting that cells expressing the toxin are resistant to toxin activity. We, therefore, tested the ability of supernatant from cultures of RJW004/pOE-B310 and boiled supernatant from cultures of an SSV9-infected strain to exogenously cause zones of inhibition on lawns of RJW004 carrying either pOE-B310, pOE-C108, pOE-B252 or pOE-Empty. Supernatants inhibited all strains except those carrying pOE-B310 (figure 2*d*). These data suggest that B310 expression is sufficient to protect the cell from inhibition by its protein product when applied exogenously and the expression of additional gene C108 did not alone protect the cell from growth inhibition.

To investigate whether B310 might be post-translationally cleaved or modified, concentrated supernatants from each overexpression strain and an SSV9 chronically infected strain were run on an SDS–PAGE gel, and slices of each gel lane were overlaid onto lawns of uninfected RJW002. Only gel lanes of the SSV9-infected and B310 overexpression strains caused zones of inhibition to form (electronic supplementary material, figure S3). These zones formed beneath the same area of each gel slice, corresponding to approximately 34 kDa, which is the predicted molecular mass of the B310 protein product. This suggests that the protein inhibits growth in a form that neither is post-translationally modified nor associates with other viral proteins.

### (c) Putative toxins from diverse SSV-infected strains

Our previous work has demonstrated that the competitive phenotype conferred by viral infection is not limited to SSV9. The CRISPR–Cas-deficient strain RJW002Δ*cas6*, when infected with SSV13 or SSV17 (isolated from Mutnovsky Volcano in Kamchatka, Russia) or SSV11 (Yellowstone National Park, USA) exhibits a killing phenotype, but not when infected with SSV14 (Kamchatka, Russia) [17]. Genome sequencing of these infected strains revealed that all except the SSV9-infected strain had independent deletions of the Type I-A CRISPR–Cas system (electronic supplementary material, table S1). SSV14 and SSV17 contain homologues of SSV9 B310. However, owing to a mutated start codon, SSV14 lacks the first approximately 60 aa, including the signal peptide, and the coding sequence begins at a downstream in-frame start codon (electronic supplementary material, figure S2). SSV11 and SSV13 do not possess obvious B310 homologues but have genes of similar size in the same orientation on their T3 transcript—p29 in SSV11 and p30 in SSV13 (electronic supplementary material, figure S2) [23].

**Figure 3.**
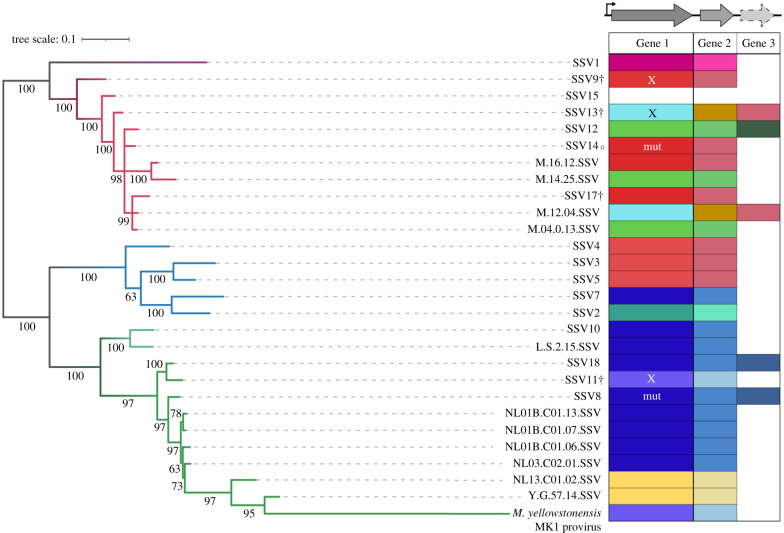
Diversity of toxin genes found among representative SSVs. Maximum-likelihood core gene tree of a subset of SSVs representing the diversity of viruses that have been isolated and sequenced to date; branches with greater than 50% bootstrap support are labelled. Branch colour represents geographical location that viruses were isolated from: Japan (purple), Kamchatka, Russia (Uzon Geyser—dark red, Mutnovsky Volcano—light red), Iceland (blue) or United States (Lassen National Park—light green, Yellowstone National Park—dark green). SSVs labelled with † have been found to confer a toxic phenotype; that marked with ○ does not [17]. Putative toxin genes upstream of the VP4 tail fibre gene were clustered using CD-HIT based on 50% identity; clusters are represented by the different colours in the table to the right of the tree. Genes with an ‘X’ were tested in this work; those marked as ‘mut’ contain a mutation that would suggest the toxin gene is non-functional, such as a mutated start codon in Gene 1 of SSV14 or a frameshift in Gene 1 of SSV8.

We compared the two to three syntenic genes on the T3 transcript from published SSVs, which were either isolated and sequenced from spring water or found as integrated elements in host genomes. Nine different alleles of possible toxin genes are present across the SSVs in this viral genomic position. Figure 3 shows these genes, along with the small adjacent one to two genes (e.g. C108 in SSV9), next to a maximum-likelihood core genome tree. Putative toxins (Gene 1) and adjacent genes (Genes 2 and 3) were grouped into types based upon homology. Amino acid alignment shows that SSV9 B310 is 56% identical to SSV14p32 (69.3% identity with 81% coverage), and has 88% identity with SSV17p29 (88.2% identity with 99.7% coverage), while SSV14p32 and SSV17p29 share 99% identity beyond the N-terminal region missing in SSV14p32 (electronic supplementary material, figure S4). Interestingly, homologues to B310 are also present in SSV3, SSV4 and SSV5 isolated from Iceland. Furthermore, divergent homologues of SSV11p29 are shared by a great many viruses from Lassen and Yellowstone, USA. The small gene(s) downstream also appear to be linked to the large gene—viruses that share the same allele of Gene 1 share Gene 2 as well (figure 3). Additionally, in the viruses that have two small genes in this position, one appears to be linked with Gene 1 while the other is not, it being found in viruses with divergent alleles of Gene 1; for example, SSV13 contains a gene homologous to SSV9 C108 (Gene 2 in figure 3).

To test whether these genes could also produce toxins we generated transcriptional profiles of SSV11, SSV13, SSV14 and SSV17 chronically infected strains (electronic supplementary material, figures S5 and S6). As shown in electronic supplementary material, figure S5, the viral coat proteins VP1 and VP3 were highly transcribed in all viruses. In addition, p29 of SSV17 (homologous to B310), as well as p29 of SSV11 and p30 of SSV13 were all highly transcribed (electronic supplementary material, figures S5 and S6). The truncated B310 homologue in SSV14, however, was not a highly expressed gene in our transcriptional analysis.

**Figure 4.**
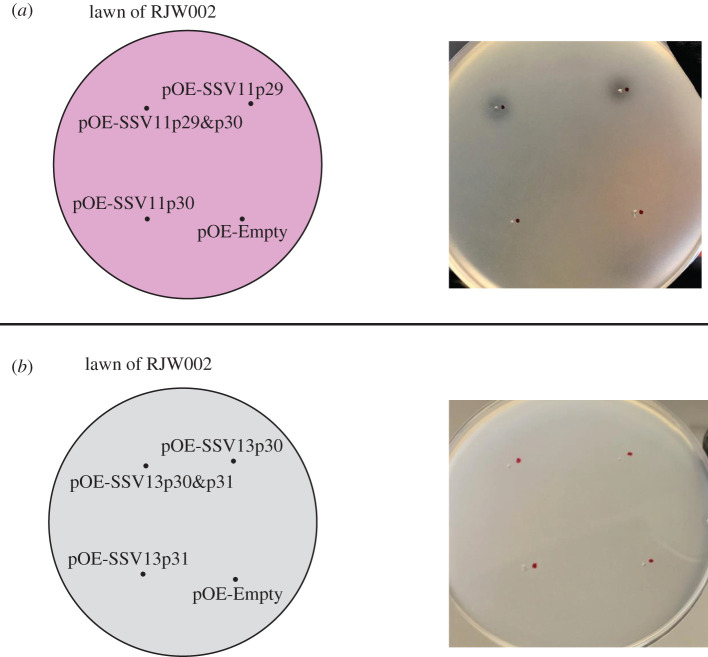
Examination of killing phenotype of putative toxins derived from SSV11 (*a*) and SSV13 (*b*). Uninfected strain RJW004 was transformed with *Sulfolobus* expression plasmid carrying both genes and each gene separately. Ten microlitres of supernatants from overexpression strains under inducing conditions was spotted on lawns of a tester strain RJW002, and then incubated at 76–78°C. Plates were imaged after 3–4 days of incubation. At least three biological replicates were set up for each sample.

Because SSV11 and SSV13 confer a toxic phenotype on hosts, and since SSV11 p29 and SSV13 p30 share the features of high expression, an N-terminal signal peptide, approximately 300 aa protein length and a common genomic location with SSV9 B310, we tested whether vector expression of these genes would produce a toxic phenotype. Arabinose-inducible overexpression vectors constructed for SSV11 were pOE-SSV11p29, pOE-SSV11p30 (short downstream gene analogous to SSV9 C108) and pOE-SSV11p29&p30; and for SSV13 were pOE-SSV13p30, pOE-SSV13p31 (short downstream gene) and pOE-SSV13p30&p31. These plasmid constructs were transformed into RJW004, and supernatant from arabinose-induced cultures of each of the resulting overexpression strains was tested for its ability to produce zones of inhibition on lawns of RJW002. As shown in figure 4, supernatant from RJW004/pOE-SSV11p29 and RJW004/pOE-SSV11p29&p30 produced zones of inhibition, but supernatant of strains with vectors encoding the SSV13 genes had no discernible effect. This is inconsistent with the data suggesting that SSV13 confers a competitive phenotype [17].

### (d) Cross-inhibition and protection among SSV toxins

**Figure 5.**
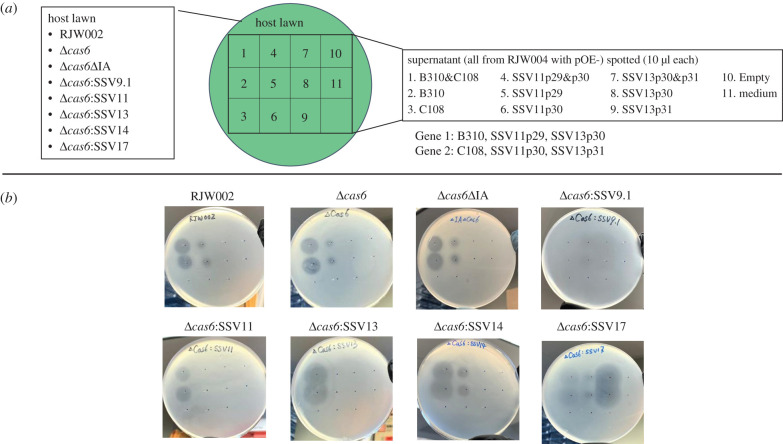
Cross-challenge of chronically infected strains among SSV toxins. (*a*) Schematic showing the lawns of different host and supernatant of RJW004 strains harbouring different plasmid constructs that were used in the spot-on-lawn assay. (*b*) Spot-on-lawn assay. Ten microlitres of collected supernatants and medium control was spotted on lawns of uninfected host (RJW002 and Δ*cas6*) and SSV-infected strains. Plates were imaged after 3–4 days of incubation at 76–78°C. The plate image shown here is a representative from three biological replicates.

The diversity of toxin genes, the possibility that the toxins themselves act as protective factors, and the combinations of additional small genes associated with each strain raised the question of whether one toxin can affect a cell that is infected by a different virus. We, therefore, cross-challenged strains infected with each SSV (SSV9, SSV11, SSV13 and SSV17) with the culture supernatants of each toxin overexpression strain. As shown in figure 5 and summarized in table 1, for each virus, the first gene (Gene 1 as shown in figure 3) is sufficient to cause growth inhibition of challenged strains while the second gene (Gene 2 as shown in figure 3) does not seem to have an effect when spotted on infected strains. Each infected cell is protected from inhibition by its own exogenously produced toxin. Supernatants from culture of strains RJW004/pOE-B310 and RJW004/pOE-B310&C108 produced zones of inhibition when spotted onto lawns of SSV11-, and SSV13- and SSV14-infected strains, low inhibition when spotted on SSV17-infected cells and no effect when spotted on SSV9-infected cells. We hypothesize that the limited effect on the SSV17-infected strain is due to the homology of its putative toxin gene with B310 (electronic supplementary material, figure S2), while the inhibiting effect on the SSV14-infected strain is due to the fact that it does not express its mutated B310 homologue. This is consistent with the finding that production of B310 is sufficient to block inhibition by the supernatant of RJW004/pOE-B310 (figure 2*d*). In addition to uninfected cell lawns, supernatant from RJW004/pOE-SSV11p29 and RJW004/pOE-SSV11p29&p30 cell cultures caused zones of inhibition on lawns of the SSV14-infected cells and low inhibition on SSV17- and SSV9-infected cells but not on SSV13-infected cells, suggesting that viruses with different toxin types can cross-inhibit each other. Surprisingly, supernatants from RJW004/pOE-SSV13p30 and RJW004/pOE-SSV13p30&p31 cell cultures inhibited the growth of SSV17- and SSV9-infected cells, despite having no effect on uninfected RJW002 and Δ*cas6* mutant, nor on the strains infected with SSV14 or SSV11. It is unclear what quality of SSV17 and SSV9 renders infected cells susceptible to SSV13p30. The complex network of cross-targeting and diversification of toxins revealed in these data suggests viral competition for hosts may occur within the hot spring environment.

**Table 1.** Summary of cross-targeting shown in figure 5. Plus and minus signs indicate presence or absence of growth inhibition. Bold indicates strong inhibition while grey indicates weak inhibition. Number of plus signs indicates relative growth inhibition.

lawn	supernatant			
	SSV9	SSV11	SSV13	Empty
RJW002	***++***	**+**	−	**−**
Δ*cas6*	**++**	***+***	−	**−**
Δ*cas6*ΔIA	**++**	**+**	−	**−**
Δ*cas6*:SSV9.1	**−**	+	++	**−**
Δ*cas6*:SSV11	**++**	**−**	**−**	**−**
Δ*cas6*:SSV13	**++**	**−**	**−**	**−**
Δ*cas6*:SSV14	**++**	**+**	**−**	**−**
Δ*cas6*:SSV17	++	+	**++**	**−**

## 3. Discussion

We used transcriptomics to identify highly expressed genes in several divergent SSVs, and by expressing these genes ectopically in uninfected strains we have shown SSV9 B310 and SSV11 p29 are the toxins responsible for the competitive phenotype of SSV-infected cells. For SSV9 B310, the toxic effect is dependent on an N-terminal portion of the gene product, absence of which (as in ‘B252’) abolishes the phenotype. This N-terminus includes a predicted Sec signal sequence (SignalP5.0) [27], which is consistent with the protein's hypothesized function as a secreted toxin. Additionally, for all the toxins experimentally tested, only host machinery is necessary for processing and for secretion, as the gene alone is sufficient to cause a toxic effect on other cells.

Growth curves of SSV9 B310 overexpression strains show that expression of this gene incurs a growth cost. Specifically, the cost of harbouring the toxin is in fact quite similar to the cost of infection that is seen in the SSV9 chronically infected strain [17]. We hypothesize that production of this secreted toxin may be responsible for the cost of infection. While the identity of an associated antitoxin is still missing, the ability of strains harbouring SSV9 B310, SSV11 p29 and SSV13 p30 to grow at all suggests that it may act as its own antitoxin or rescue factor, as no other viral genes are present to fill that role. We hypothesize that the toxin is processed as it is secreted from the cell, and this processed toxin then shows activity from without. This has been seen before in killer yeast toxins, in which the preprocessed toxin acts as its own immunity factor within the infected cell [28]. Furthermore, the function of the linked two downstream genes associated with the toxins is not clear from these studies. We hypothesize that they may have some function in the context of the viral particle owing to the proximity to the tail fibres.

In gene expression studies of SSVs, genes syntenic to the B310-like genes, on the T3 transcript, are highly expressed [23]. For both SSV1 and SSV2, the gene in this position is among the most highly expressed genes in the non-induced or carrier state of infection [13,14,16], consistent with our results for the viruses used in the present study. During induction or naive infection, this gene was expressed with other late genes like the viral coat proteins in SSV1 but was the first gene expressed with SSV2. It has been previously hypothesized that this gene plays a role in virus docking and release owing to the expression at the same time as the viral coat proteins and the presence of a signal sequence and transmembrane domain in the SSV2 gene [16]. While the genes identified as encoding toxins in this study do have signal sequences, they do not contain transmembrane domains; however, the short downstream genes do. It should be noted here that SSV1 and SSV2 have unique alleles in this position when compared with those tested in this study (figure 3).

The ability of both chronically infected and toxin overexpression strains to inhibit and kill other uninfected cells perhaps explains the anomalous plaquing phenotype that has been seen with SSVs. Since SSVs have been shown to egress from the cell in a non-lytic manner, by budding, the ability of SSVs to form plaques on lawns of cells has long been an open question [18,20,29,30]. One possibility is that the plaquing phenotype is not due to any cellular lysis, but rather to the action of the toxin: once a cell becomes infected with an SSV and begins to produce toxin, it will form a clearing around itself as it kills neighbouring cells. This would also be consistent with the lack of growth seen in naive infections with SSV9, as well as the dormancy phenotype of cells briefly challenged with viral particles [29,30]. The difference in inhibition shown by the spot-on-lawn assay in figure 5 is consistent with dormancy phenotype, which we have previously shown leads to cell death when exposure to supernatant from infected cells is prolonged [29]. Therefore, it is likely that this growth inhibition could lead to killing of uninfected strains when they compete with infected strains in co-culture [17].

The interactions shown in figure 5 suggest several intriguing potential mechanisms of inhibition and protection that are dependent on which variant of the chronic virus is infecting each host. In general, it appears that intracellular expression of the toxin protects cells from exogenous activity of the same toxin. We suggest that the toxin is modified or cleaved in some way, though its molecular mass in supernatant is similar to what is predicted based on the amino acid sequence. There are examples among the archaea of a toxin acting as its own protective factor—for example, halocin C8, a toxin produced by certain strains of haloarchaea, is cleaved into a short secreted toxic peptide and a large immunity protein [31]. In addition, this protection is possibly allele-specific, as indicated by the cross-targeting of strains carrying different alleles. The strong toxicity of SSV13 p30 against SSV17-infected cells, while it had no effect on uninfected cells, indicates mediation of the interaction through alternate virus infection. This SSV17-infected strain has also deleted its Type I-A CRISPR–Cas system, meaning that we cannot exclude an impact of CRISPR–Cas on susceptibility to these toxins. To generate chronically infected strains, we used a strain of *S. islandicus* with *cas6*, which codes for the key crRNA (CRISPR RNA) processing enzyme, deleted. It is unknown why these strains independently deleted the Type I-A CRISPR region unless activated Cascade (CRISPR-associated complex for antiviral defense) is toxic to the cells in the absence of crRNAs. The data provided in figure 5 suggest that deletion of the Type I-A cascade does not impact sensitivity of our type strain to toxin.

Hot springs can contain many strains of both *S. islandicus* [32–34] and SSVs [35]; it is possible that divergent host and viral strains could compete via virus-encoded toxins. In previous work, we demonstrated the competitive advantage of chronically SSV-infected strains over other host types, such as CRISPR–Cas immune strains. We previously hypothesized that this phenotype evolved in Kamchatka under conditions of high distributed CRISPR–Cas immunity where susceptible hosts are rare as a strategy to promote viral fitness through vertical transmission [17,35–39]. Given the metapopulation structure seen in hot spring locations, the killing phenotype would theoretically benefit the host not only in invasion of a new hot spring environment but also in helping it to maintain dominance in an already established population [17,34,40]. However, the present work highlights the potential for competitive dynamics between host strains chronically infected with different viruses. This would suggest that the basis of this mutualism, at least in some populations, could be competition between the viruses to promote the growth of their hosts and enhance vertical transmission. The cross-toxicity network appears to represent a nested structure in which some viral toxins are potent against all strains (SSV9) while others are only toxic against a few. Such networks of interactions are hypothesized to maintain stability and diversity in mutualisms [41].

## 4. Material and methods

### (a) Strains and growth conditions

All *S. islandicus* strains (electronic supplementary material, table S2) were grown in dextrin–EZMix N-Z-Amine (DT) medium at pH 3.5 and supplemented with 20 µg ml^−1^ of uracil (U) and 50 µg ml^−1^ of agmatine (A) when required as previously described [42]. Cultures were incubated in vented tissue culture flasks (Fisher Scientific, USA) without shaking at 76–78°C. Plates used for spot-on-lawn assay were made using sucrose–yeast (SY) medium with 1.4% Gelrite (w/v) for bottom layer and 0.8% (w/v) Gelrite for top layer.

### (b) RNA extraction and sequencing

Biologically triplicate cultures of uninfected (Δ*cas6*) and chronically infected strains (Δ*cas6*:SSV9.1, Δ*cas6*:SSV11, Δ*cas6*:SSV13, Δ*cas6*:SSV14 and Δ*cas6*:SSV17) were grown to mid-log phase (OD_600 nm_ = 0.20–0.24) in DTU medium. Cells were collected by pelleting and flash frozen in ethanol and dry ice. Total RNA was prepared with a QIAGEN RNeasy kit with on-column DNase treatment with the QIAGEN RNase-free DNase Set according to the manufacturer's protocols. RNA was visualized by agarose gel electrophoresis and quantified with a Qubit 2.0 fluorometer and Qubit RNA BR Assay Kit (Thermo Fisher Scientific, USA). Samples were stored at −80°C before submission to the W. M. Keck Center for Comparative and Functional Genomics at the University of Illinois at Urbana-Champaign (UIUC) for RNA sequencing (RNAseq). rRNA depletion was carried out with the RiboZero bacterial kit (Illumina, USA) and libraries were prepared with the TruSeq Stranded mRNAseq Sample Prep Kit (Illumina, USA). Libraries were quantified by qPCR and sequenced on a NovaSeq600. FastQ files were generated and demultiplexed using bcl2fastq v. 2.20 conversion software (Illumina, USA). Reads were trimmed with Cutadapt and quality filtered using Sickle [43,44].

### (c) Viral expression analysis

To elucidate which viral genes are highly transcribed, expression levels of viral transcripts were determined using Rockhopper [45]. Trimmed and quality-filtered reads were mapped to each respective viral reference genome as well as the *S. islandicus* M.16.4 host genome using default parameters. Expression levels for each transcript were determined in Rockhopper by number of reads mapped divided by the gene's length and divided by gene expression in the upper quartile. Expression levels are representative of all three biological replicates together.

### (d) DNA extraction and sequencing

To confirm the infection with full length, wild-type viruses, chronically infected strains were sequenced and analysed for mutations. Chronically infected strains were grown to mid-log phase and genomic DNA was extracted using phenol–chloroform and isoamyl alcohol purification as previously described [40,46]. Libraries were prepared with the Kappa Biosystems Hyper Library construction kit (Roche, USA). Libraries were quantified by qPCR and sequenced on a NovaSeq600. FastQ files were generated and demultiplexed using bcl2fastq v. 2.20 conversion software (Illumina, USA). Reads were trimmed with Cutadapt and quality filtered using Sickle [43,44]. Genomic mutations were determined by *breseq* using default parameters [47].

### (e) Overexpression of SSV genes and mutant construction in *Sulfolobus islandicus*

SSV genes of interest were either PCR-amplified from viral genomic DNA with the primers listed in electronic supplementary material, table S3 or synthesized by Integrated DNA Technologies (electronic supplementary material, table S4), and then cloned into the *Sulfolobus* replicative vector pSeSd-SsoargD ([48]; hereafter named pOE-Empty), which contains an arabinose-inducible promoter. The resulting recombinant plasmids were delivered into host strain *S. islandicus* RJW004 via electroporation-mediated transformation as described previously [42], generating strains that expressed toxin-, antitoxin- or toxin/antitoxin-candidate genes. A mutant strain lacking *cas6* and Type I-A CRISPR–Cas module (*cas3b*, *csa5*, *cas7*, *cas6*, *cas3*′, *cas3*″ and *csaX*) was constructed in *S. islandicus* RJW007 via a so-called plasmid integration and segregation approach, as previously described [42].

### (f) Growth curves

Overexpression strains and controls were grown in sterile tissue culture flasks containing 50 ml of DT or DTUA medium to exponential phase. Cell cultures were harvested, washed three times by centrifugation at 3220*g* for 15 min, and resuspended with carbon-source-free medium. The optical density of resuspended cells was normalized to OD_600 nm_ = 0.5, and then the cells were transferred into 50 ml of 0.2% arabinose (w/v) medium for inducing conditions, adjusting the initial OD_600 nm_ of all samples to 0.02. Growth was monitored every 24 h for 5 days by measuring optical density with a WPA CO8000 Cell Density Meter (Biochrom, USA).

### (g) Testing killing activity and cross-challenge of chronically infected strains

Supernatants from RJW004 carrying plasmid constructs of toxin-, antitoxin- and toxin/antitoxin-candidates were collected from exponentially growing cultures (OD_600 nm_ = 0.12–0.2) by centrifuging at 3220*g* for 15 min, and were then filtered with 0.2 µm Sterile Disposable Vacuum Filter Units (Fisher Scientific, USA) and stored at 4°C until use.

Killing activity of collected supernatants was determined by spot-on-lawn tests. Lawns of the tester strain RJW002 (*S. islandicus* M.16.4 Δ*pyrEF*) were concentrated to 10× by centrifuging at 3220*g* for 15 min, and 500 µl of concentrated cells was plated with 5 ml of overlay medium (2.5 ml of 0.8% Gelrite and 2.5 ml of 2 × SY medium) onto SY plates supplemented with uracil as needed. Ten microlitres of each supernatant was spotted onto lawns and allowed to dry before plates were bagged and incubated at 76–78°C for 3–4 days.

Similarly, for the cross-challenge, uninfected (RJW002 and Δ*cas6* mutant) and chronically infected strains (Δ*cas6*:SSV9.1, Δ*cas6*:SSV11, Δ*cas6*:SSV13, Δ*cas6*:SSV14 and Δ*cas6*:SSV17) were grown in fresh medium to exponential phase. The lawns of these strains were prepared as described above and then spotted by 10 µl of collected supernatant. The presence and absence of plaques were recorded and imaged after 3–4 days of incubation at 76–78°C.

### (h) In-gel killing activity assay

To further support toxin identification and investigate potential post-translational modifications, supernatants from chronically infected strains and overexpression strains were analysed by protein gel electrophoresis and killing activity. Supernatants were collected from uninfected, chronically infected or overexpression strains and concentrated using tangential flow filtration as previously described [17]. Concentrated supernatants were quantified for total protein using the Pierce BCA assay kit and then normalized. Supernatants were then mixed with 2 × Laemmli sample buffer containing β-mercaptoethanol and heated at 95°C for 10 min. Samples were run at 115 V for 60 min on a Mini Protean TGX 4–20% gel with Precision Plus Protein Kaleidoscope Standards in a BioRad mini gel set-up with 1 × Tris–glycine–SDS buffer. Gels were visualized with the Pierce Silver Stain Kit according to the manufacturer's instructions. Gels to be plated were rinsed three times for 15 min with Milli-Q water before being cut into lanes with a fresh razor. Lanes were then placed into 5 ml of freshly poured, still liquid overlay medium with 500 µl of 10× concentrated RJW002 cultures on SYU plates. After the initial lawns solidified with the protein gel lanes, another 5 ml of overlay medium with concentrated culture was poured on to completely cover the protein gel. Plates were then bagged and incubated for 3 days at 76–78°C.

### (i) Comparison of spindle-shaped viruses and their toxin genes

Open reading frames from a subset of SSVs were obtained from NCBI and core genes were identified and clustered using CD-HIT [49,50] with a sequence identity cut-off of 0.4. Genes thus identified were the homologues of SSV1 VP1, VP3, VP4, B277, B129, A153, A82, B78, C166, B115, A92 and C84 [51]. The nucleotide coding sequences of the respective gene clusters were aligned using MAFFT v. 7 [52], concatenated, and used to generate a phylogenetic tree with RAxML [53] with settings -f a -x 100-p 100 -N autoMR -m GTRGAMMA [35]. Putative toxin genes were determined by sequence similarity to SSV9, SSV11, SSV13, SSV14 or SSV17 sequences or determined by proximal location to the VP4 tail fibre gene, downstream of the T3 promoter. Homologous toxin genes were determined using CD-HIT with a sequence identity cut-off of 0.5 [49,50]. Homologous toxin genes were aligned using MAFFT and manually inspected [54]. Toxin genes that clustered together were then translation aligned using Geneious Prime 2020.2.4 and d*N*/d*S* was calculated using the SNAP online tool (www.hiv.lanl.gov).

## Data accessibility

The RNA-Seq raw reads generated in this work have been deposited into the NCBI SRA database under the accession number PRJNA750755. The strains and plasmids generated in this study are available from the corresponding author upon a reasonable request.

The data are provided in the electronic supplementary material [55].
